# Monoclonal antibody targeting the conserved region of the SARS-CoV-2 spike protein to overcome viral variants

**DOI:** 10.1172/jci.insight.157597

**Published:** 2022-04-22

**Authors:** Wan-Ling Wu, Chen-Yi Chiang, Szu-Chia Lai, Chia-Yi Yu, Yu-Ling Huang, Hung-Chun Liao, Ching-Len Liao, Hsin-Wei Chen, Shih-Jen Liu

**Affiliations:** 1 National Institute of Infectious Diseases and Vaccinology, National Health Research Institutes (NHRI), Miaoli, Taiwan.; 2 Institute of Preventive Medicine, National Defense Medical Center, New Taipei City, Taiwan.; 3 Department of Life Sciences, National Tsing Hua University, Hsinchu, Taiwan.; 4 Graduate Institute of Biomedical Sciences, China Medical University, Taichung, Taiwan.; 5 Graduate Institute of Medicine, College of Medicine, Kaohsiung Medical University, Kaohsiung, Taiwan.

**Keywords:** COVID-19, Therapeutics, Drug therapy

## Abstract

Most therapeutic mAbs target the receptor-binding domain (RBD) of the spike protein of SARS-CoV-2. Unfortunately, the RBD is a hot spot for mutations in SARS-CoV-2 variants, which will lead to loss of the neutralizing function of current therapeutic mAbs. Universal mAbs for different variants are necessary. We identified mAbs that recognized the S2 region of the spike protein, which is identical in different variants. The mAbs could neutralize SARS-CoV-2 infection and protect animals from SARS-CoV-2 challenge. After cloning the variable region of the light chain and heavy chain, the variable region sequences were humanized to select a high-affinity humanized mAb, hMab5.17. hMab5.17 protected animals from SARS-CoV-2 challenge and neutralized SARS-CoV-2 variant infection. We further identified the linear epitope of the mAb, which is not mutated in any variant of concern. These data suggest that a mAb recognizing the S2 region of the spike protein will be a potential universal therapeutic mAb for COVID-19.

## Graphical abstract

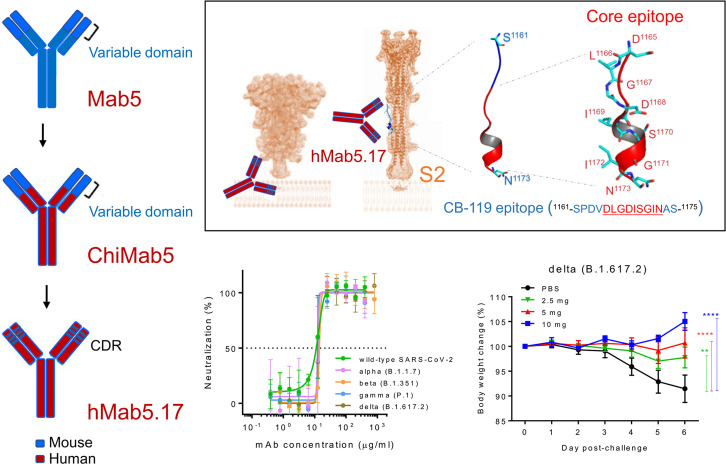

## Introduction

The COVID-19 outbreak is an emerging global health threat, and the virus continues to spread worldwide. SARS-CoV-2, a betacoronavirus, is the major cause of COVID-19 (1, 2). The total number of COVID-19 cases exceeded 250 million infections and 5 million deaths as of November 10, 2021 (3). Even though COVID-19 vaccines are now available to prevent illness for most of the world, the epidemic is still increasing sharply in many parts of the world, with little sign of slowing. Thus, the rapid development of safe and effective new drugs for COVID-19 treatment is of particular concern. Among possible interventions, the efficacy of passive immunization with convalescent plasma to cure COVID-19 has been proven, resulting in clinical improvement (4, 5); therefore, the development of neutralizing mAbs is one of the most promising approaches for clinical use and can complement the use of vaccines to block infection in patients with SARS-CoV-2 (6).

SARS-CoV-2 uses its spike (S) glycoprotein to bind the human angiotensin-converting enzyme 2 (hACE2) receptor and mediate membrane fusion for virus entry (7–10). The S protein comprises an N-terminal S1 subunit, in which the receptor-binding domain (RBD) contains the major epitopes eliciting the production of neutralizing Abs (11), and a C-terminal S2 subunit containing 2 heptad repeat (HR) regions (HR1 and HR2) that facilitate fusion with the cell membrane (8, 12). Current vaccine strategies using the S protein to induce the production of neutralizing Abs are an important component contributing to the protection of humans against SARS-CoV-2 infection (8, 13). However, numerous reported neutralizing mAbs targeting the RBD to block the interaction with ACE2 fail to neutralize many of the clinical variants because multiple mutations occur in the highly variable RBD, allowing the virus to escape neutralization (14–16). Indeed, the recently emerged lineages of viral variants in the United Kingdom (B.1.1.7), South Africa (B.1.351), and Brazil (P.1) with mutations in the RBD exhibit resistance to neutralizing mAbs and have reduced the efficacy of vaccines and immunity from natural infection (17–19). Therefore, much effort is still required to develop therapeutic mAbs or vaccines eliciting the production of broadly neutralizing Abs against evolving variants through engagement with a more conserved, cryptic epitope on the spike protein.

Because some regions within the S1 subunit have greater sequence diversity, the highly conserved S2 among coronaviruses may be beneficial for therapeutic interventions (20, 21). Some molecules and peptides targeting the highly conserved HR regions of S2 have been reported to be effective against SARS-CoV-2 as well as other human coronavirus (hCoV) infections by blocking S2-mediated membrane fusion and pseudovirus transduction (20, 22, 23). However, only a few anti-S2 neutralizing mAbs against human-infecting coronaviruses have been hitherto characterized, and it is even rarer that such mAbs have been characterized for their neutralizing ability against emerging variants (Table 1) (24–31). Among them, 2 SARS-CoV-2-neutralizing mAbs, CC40.8 and CV3-25, which were isolated from patients with COVID-19, showed weak cross-neutralization against SARS-CoV-1, and 1 demonstrated a neutralizing response against SARS-CoV-2 variants (24, 26, 30). Based on the aforementioned studies, the identification of neutralizing mAbs targeting epitopes in the highly conserved S2 subunit is tenable for the design of intervention strategies against coronavirus-associated diseases.

In this study, we identified 2 neutralizing mAbs described in our previous study (32), termed Mab5 and Mab3-2, whose production was elicited by SARS-CoV-1 and that caused serious illness and death in the 2002–2003 outbreak and now cross-react with SARS-CoV-2 by binding to the conserved region on the S2 subunit. Phylogenetically, the 2 viruses share approximately 77% amino acid sequence identity in the whole S protein and 90% identity in S2 (33). Using the Syrian hamster model, we assessed the in vivo therapeutic activity of 2 mAbs against SARS-CoV-2. Furthermore, a promising candidate, murine Mab5, with highly potent neutralizing activity, was chosen for humanization. We also demonstrate that humanized Mab5 (hMAb5) possesses strong neutralizing ability and a protective effect in vivo against SARS-CoV-2 and displays broad neutralizing potency against emerging SARS-CoV-2 variants of concern (VOCs). Herein, we demonstrate for the first time to our knowledge, through in vitro neutralization assays and in vivo protection experiments that 1 anti-S2 neutralizing mAb against SARS-CoV-1 can cross-react with SARS-CoV-2 and even cross-neutralize emerging SARS-CoV-2 variants.

## Results

### Two mAbs recognize the S2 subunit of SARS-CoV-2.

**Figure 1:**
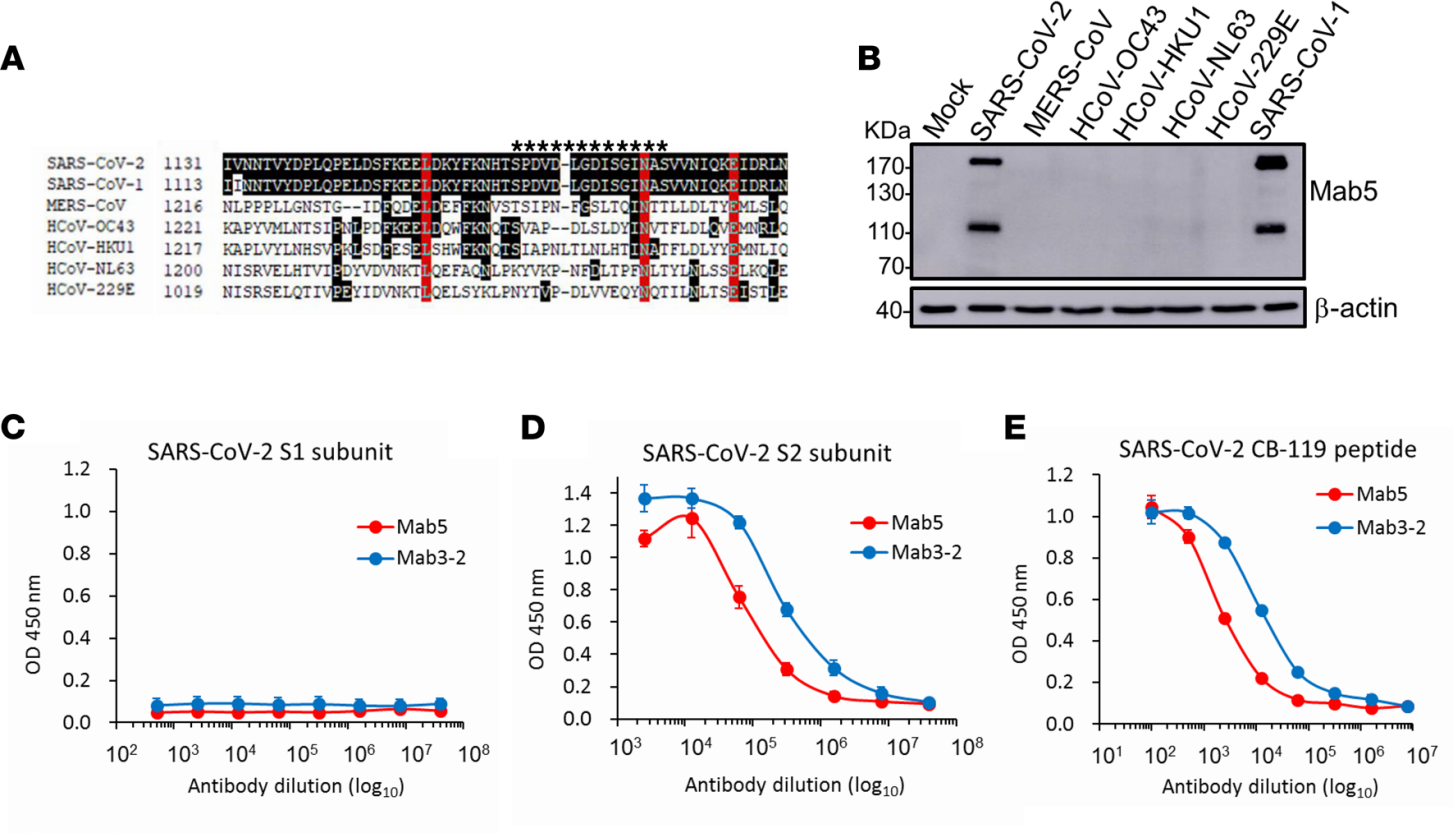
mAbs recognized the S2 subunits of both SARS-CoV-1 and SARS-CoV-2. (**A**) Multiple sequence alignments of infectious hCoV HR2 regions, including those of SARS-CoV-2, SARS-CoV-1, Middle East respiratory syndrome–related coronavirus (MERS-CoV), and HCoV-OC43, HCoV-HKU1, HCoV-NL63, and HCoV-229E. The CB-119 epitope residues are marked by asterisks. The residues are numbered according to their positions on the SARS-CoV-2 S protein sequence. The red positions represent identical residues, and the black shading indicates highly conserved residues among these sequences. (**B**) Expression plasmids encoding S proteins from the aforementioned strains were transiently transfected into 293T cells. Subsequently, S protein from each cell lysate was detected by immunoblotting using mAbs. β-actin represents the internal control of each lysate. See complete unedited blots in the supplemental material. (**C**–**E**) The binding efficacy of the Mab5 and Mab3-2 mAbs for the (**C**) recombinant S1 and (**D**) S2 subunits of SARS-CoV-2 and (**E**) synthetic peptide CB-119 was determined by antigen-coating ELISA.

Several neutralizing mAbs that sufficiently inhibit SARS-CoV-1 infection previously were identified (32). Among these mAbs, 2 strong neutralizing mAbs, Mab5 and Mab3-2, targeting the highly conserved HR2 domain were selected to assess the cross-reactive response with SARS-CoV-2. We first performed S protein sequence alignment for SARS-CoV-1 and SARS-CoV-2, and found that the C-terminal region of the S2 subunit is highly conserved, especially with 100% identity in the HR2 domain and neutralizing epitope (CB-119) of the Mab5 and Mab3-2 Abs (Supplemental Figure 1A; supplemental material available online with this article; https://doi.org/10.1172/jci.insight.157597DS1). Consistent with the alignment results, 2 SARS-CoV-1–specific mAbs cross-reacted strongly with the S protein of SARS-CoV-2 (Supplemental Figure 1B). To further examine the cross-reactive binding of the 2 selected mAbs against other human-infecting coronaviruses, a sequence focusing on the residues of the CB-119 epitope was aligned (Figure 1A), and individual S proteins were expressed to determine Ab binding by immunoblot analysis. As expected, the SARS-CoV–specific mAbs consistently cross-recognized only the S protein of SARS-CoV-2 but not that of other related coronaviruses (Figure 1B). Furthermore, the binding activities of the mAbs were also determined by ELISA using purified S2 subunit protein (Figure 1D) and synthetic peptide CB-119 as antigens (Figure 1E), but no binding was observed with the S1 subunit (Figure 1C). These observations clearly indicated that the 2 mAbs have the potential to neutralize SARS-CoV-2 through specific recognition of CB-119 located in the HR2 region of the spike protein.

### Neutralization activity and binding kinetics of the mAbs.

**Figure 2:**
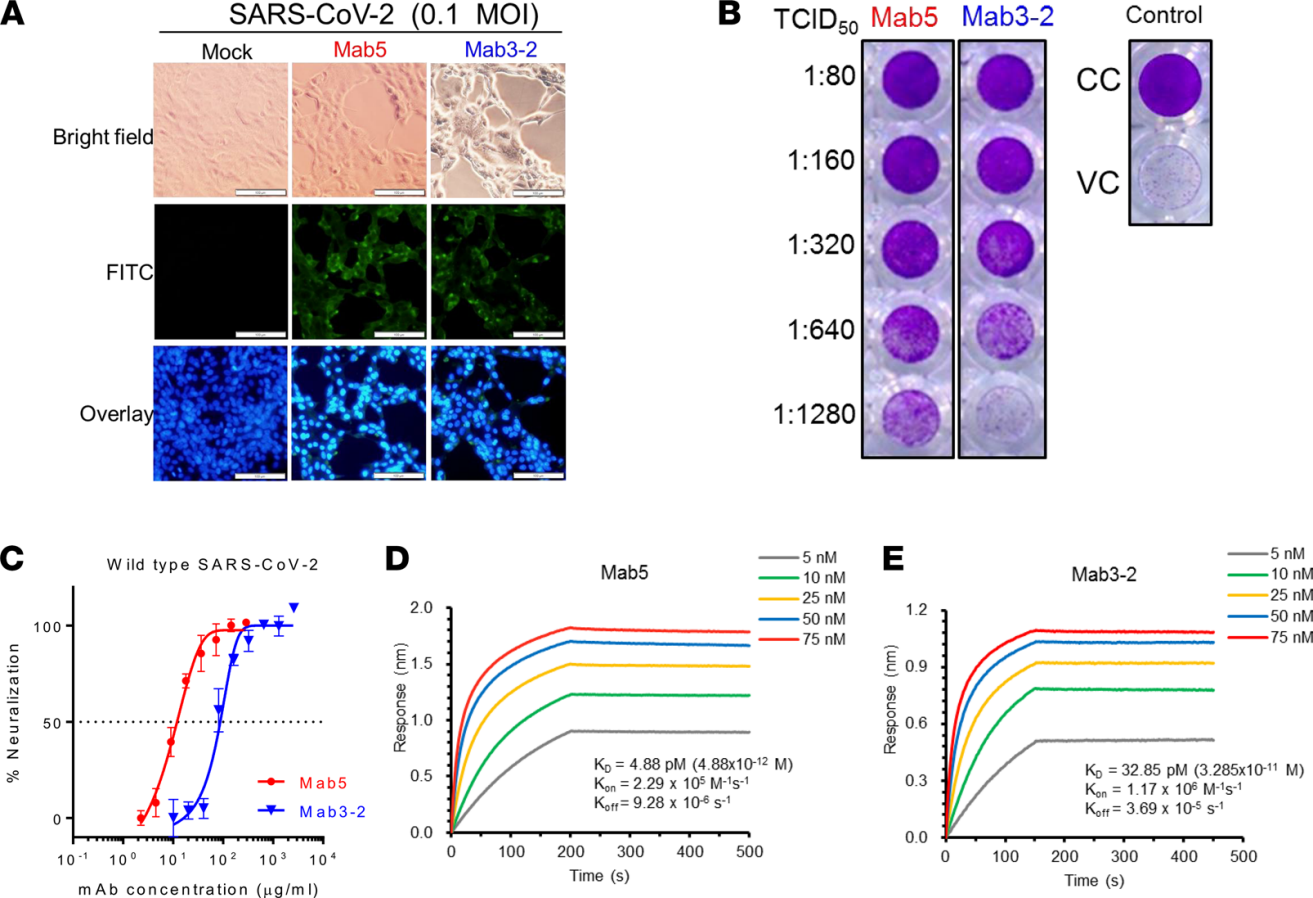
Neutralization potency and binding affinity of mAbs. (**A**) The binding of mAbs to Vero cells infected with SARS-CoV-2 (0.1 MOI) was detected by IFA. Vero cells were cultured in 24-well plates and infected with SARS-CoV-2 for 1 day. For IFA, mock control cells without the primary mAb and cells were treated with the indicated primary Abs and probed with FITC-conjugated goat anti–mouse IgG secondary Abs. The cell nuclei in the overlay images were visualized by DAPI staining (blue). Scale bar: 100 μm. (**B**) The TCID_50_ neutralization assay results were visualized by 0.5% crystal violet staining. (**C**) The neutralization efficiency of Mab5 and Mb3-2 against authentic SARS-CoV-2 was evaluated by calculating the percentage of neutralization. The horizontal dotted line indicates 50% neutralization. (**D** and **E**) The kinetics of (**D**) Mab5 and (**E**) Mab3-2 binding to the S2 subunit were measured by BLI with antigens on the biosensor and Abs in solution. Representative results from 2 replicates of each experiment are shown. Representative results from 1 of 2 replicated experiments with similar results are shown. CC, cell control without viral infection; VC, virus control without Ab treatment.

Having identified mAbs against the SARS-CoV-2 S protein under denaturing conditions, we performed an immunofluorescence assay (IFA) to investigate whether the mAbs recognize the natural form of the S protein in SARS-CoV-2–infected cells. The binding capability could be visualized by a distinct fluorescence signal when the virus-infected Vero cells were treated with Mab5 or Mab3-2, whereas no signal was observed in the mock control cells (Figure 2A).

To further explore the neutralization activity of the mAbs against SARS-CoV-2, 2-fold serial dilutions of individual mAbs ranging from 1:80 to 1:1280 were quantified by end-point titration on Vero cells through the cytopathic effect–based (CPE-based) 50% tissue culture infectious dose (TCID_50_) method. The status of virus-induced CPE was monitored, and the percentage of neutralization was further calculated on the basis of a colorimetric assay with crystal violet staining. The results provided clear evidence that the 2 mAbs possessed neutralizing ability against SARS-CoV-2, with an IC_50_ value of 12.3 μg/mL for Mab5 indicating better neutralizing activity than the IC_50_ of 87.4 μg/mL for Mab3-2 (Figure 2, B and C). Of note, their binding affinities, determined by biolayer interferometry (BLI), showed that Mab5 bound much more tightly than Mab3-2 to the S2 subunit, with dissociation constants (*K_D_*) of 4.88 pM and 32.85 pM, respectively (Figure 2, D and E).

These results reflect that the binding affinity of the mAbs to the S protein is well correlated with the ability to neutralize SARS-CoV-2. Our findings indicated that both mAbs were able to cross-neutralize SARS-CoV-2; however, Mab5 was more potent than Mab3-2, because of its strong neutralizing potency.

### Protective efficacy of the mAbs against SARS-CoV-2 infection in Syrian hamsters.

**Figure 3:**
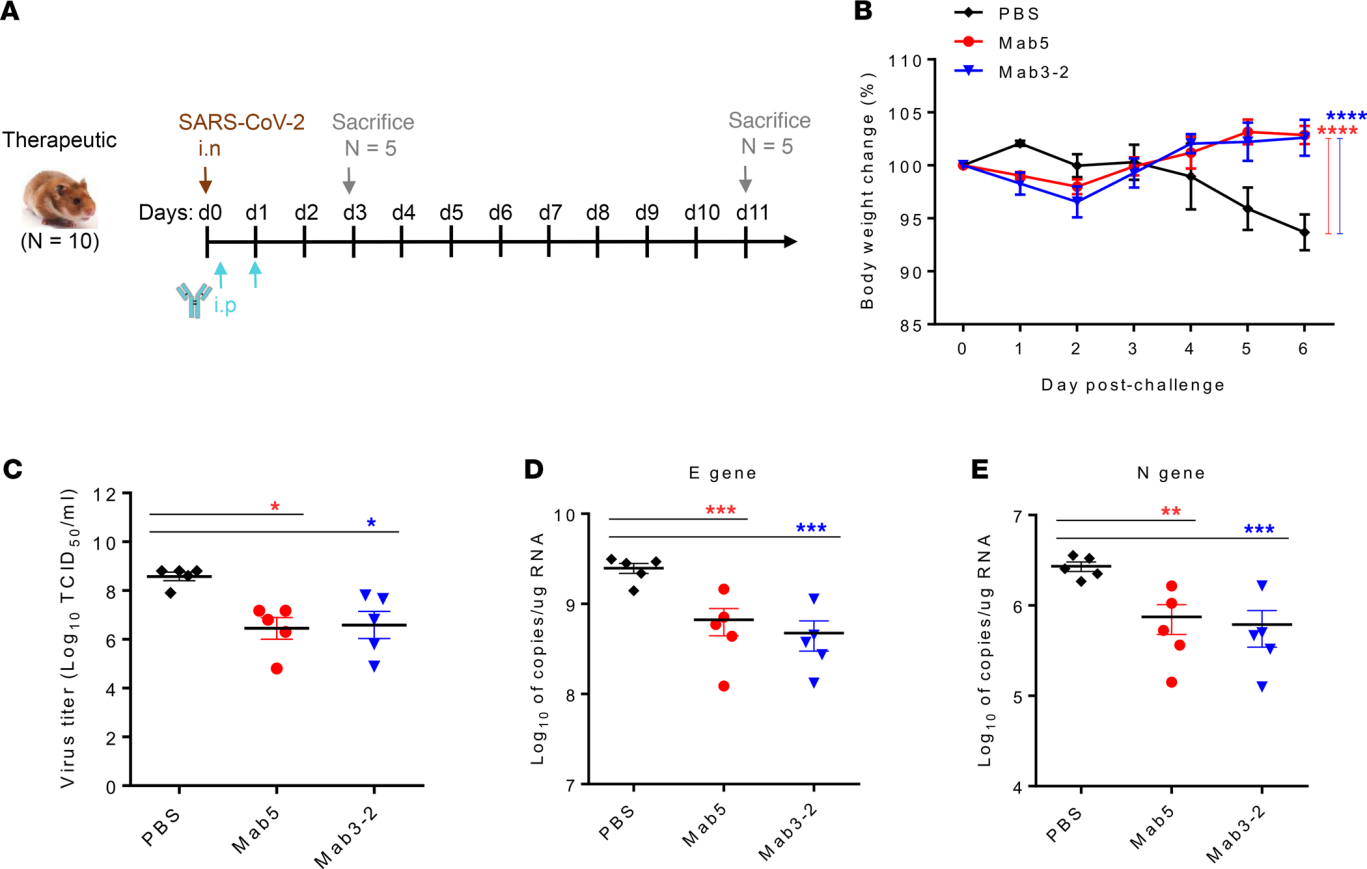
Protective efficacy of neutralizing mAbs against SARS-CoV-2 infection in Syrian hamsters. (**A**). SARS-CoV-2 challenge model for determining the therapeutic efficacy. Each group (*n =* 10) was challenged intranasally with 10^5^ TCID_50_ of SARS-CoV-2. Each hamster was intraperitoneally injected with 2.5 mg (16.5 mg/kg) of neutralizing mAb at 3 hours and again at 1 day after infection, and in parallel, control hamsters were injected with saline. The percent body weight change was recorded over 11 days. (**B**) For determination of the therapeutic efficacy of murine mAbs, the animals were treated with Abs by intraperitoneal injection at 3 hours and again at 24 hours after infection. The percent body weight change was recorded daily over 11 days. (**C**) The infectious viral load in the lung tissues (*n =* 5) on day 3 was quantified by TCID_50_ assay. (**D** and **E**) The viral loads were determined by RT-qPCR targeting 2 SARS-CoV-2 genes (labeled **E** and **N**). The data were statistically analyzed by (**B**) 2-way ANOVA followed by Tukey multiple-comparison test and (**C**–**E**) ordinary 1-way ANOVA followed by Tukey multiple-comparison test. All data are reported as the mean ± SEM. *P < 0.05, **P < 0.01, ***P < 0.001, and ****P < 0.0001 compared with the control.

To further ascertain the efficacy of Ab-based protection against SARS-CoV-2 in vivo, we applied a Syrian hamster animal model that was established by challenge with 10^5^ TCID_50_ of virus. For the experimental design, shown in Figure 3A, the animals were administered a single dose of 2.5 mg (16.5 mg/kg body weight) mAb via passive intraperitoneal transfer at 3 hours and again at 1 day after infection, and all the animals were monitored daily for body weight changes. For viral RNA and infectious virus quantification assays, half of the animals from each group (*n =* 5) were sacrificed 3 days after virus exposure, and lung tissues were collected. Daily weight-change data indicated that animals from the Mab5 and Mab3-2 treatment groups maintained their body weight, whereas the negative control group lost a significant amount of weight (Figure 3B). Furthermore, hamsters treated with mAbs exhibited significantly decreased infectious virus titers (Figure 3C) and reduced active virus replication, based on viral RNA levels (Figure 3, D and E), relative to those of the control hamsters. Together, these data demonstrated that the 2 neutralizing mAbs had a protective effect in vivo against SARS-CoV-2 infection.

### Engineering recombinant chimeric and hMAb5 for functional characterization.

Aiming to minimize the immunogenicity and toxicity of therapies against SARS-CoV-2 infection in humans, we selected an excellent candidate, murine Mab5, for chimerization and further humanization. By genetically fusing the heavy chain variable regions (VHs) and light chain variable regions (VLs) of murine Mab5 to human Ab constant regions, a chimeric Mab5 (ChiMab5) was successfully expressed and purified from CHO cells (Supplemental Figure 2A). The results showed that ChiMab5 retained neutralization capacity against SARS-CoV-2 and binding affinity comparable with those of its parental Ab (Supplemental Figure 2, B and C).

To develop much less immunogenic and more specific Abs than ChiMab5 for therapeutic uses, murine complementarity-determined regions were grafted into a homologous human framework template to generate an hMAb5 (Supplemental Figure 3A). IgBLAST analysis (34) showed that Ig HV and Ig κ chain variable regions of murine Mab5 have 7 and 4 amino acid changes (somatic mutations), respectively, during affinity maturation from the original germline Ab sequence. The closest human germline sequences identified as templates for humanization were IGHV3-23*04 and IGKV4-1*01 (Supplemental Table 1 and Figure 3B). Moreover, we introduced 7 canonical residues of back mutations in VH and 5 canonical residues in VL into the human framework templates and designed 5 versions of back-mutated VHs (named VH1, VH2, VH3, VH4, and VH5) and 2 versions of back-mutated VLs (VL1, VL2, VL3, and VL4) (Supplemental Figure 3A and Supplemental Table 2). All versions of VHs and VLs were combined to make 20 humanized Abs.

To rapidly evaluate the binding affinity and neutralization activity of humanized Abs in preliminary screening, the 20 constructs were transiently transfected into cells, and the expressed supernatants were collected to confirm high potency. We identified 9 mAbs with improved antigen-binding affinities and neutralizing potency to block authentic SARS-CoV-2 CPE production (Supplemental Table 2). In other words, the 9 humanized Abs possessed the antigen-binding specificity and neutralization capacity most similar to those of their chimeric analog.

**Figure 4:**
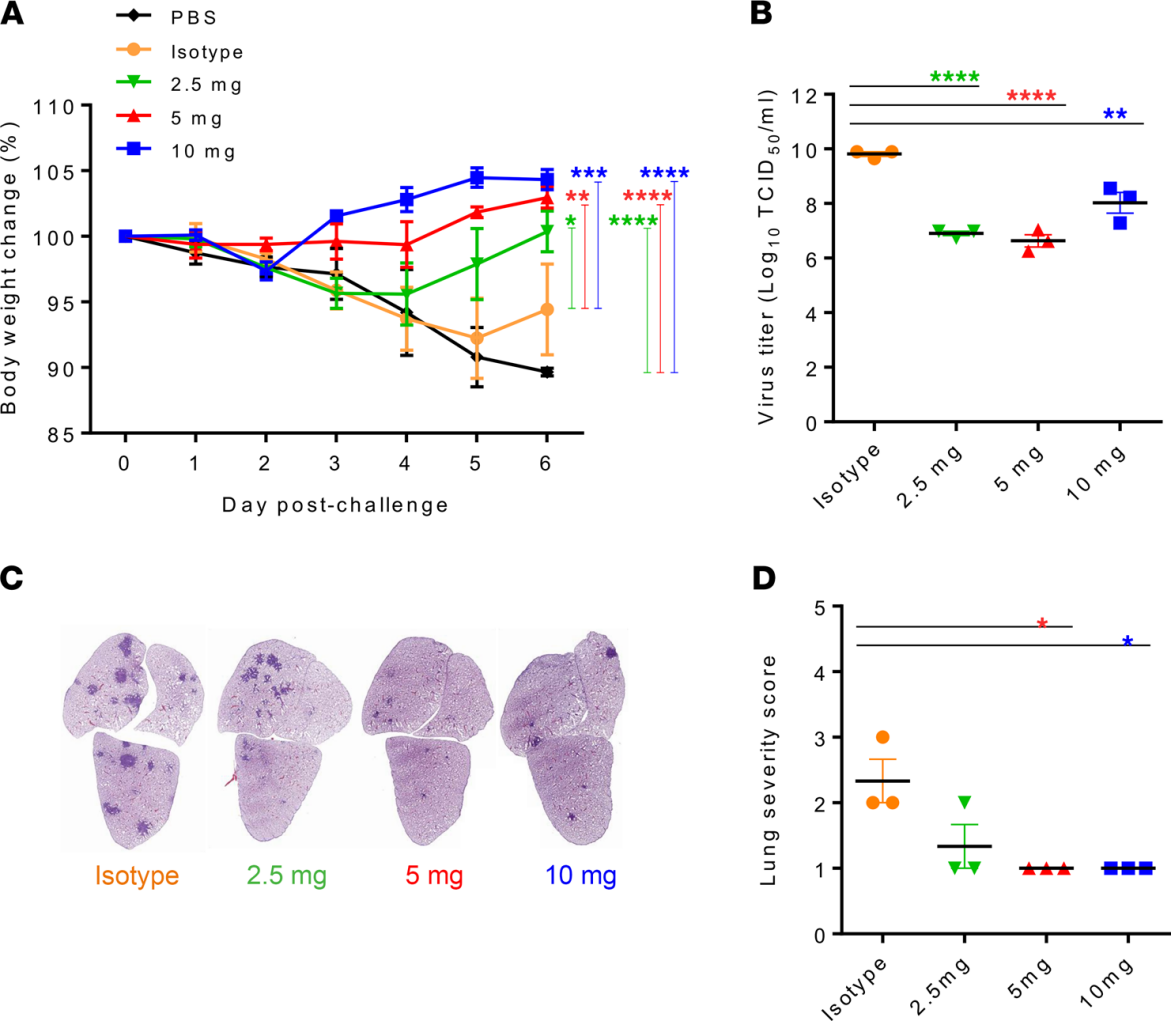
Therapeutic efficacy of hMab5.17 against SARS-CoV-2 infection in Syrian hamsters. (**A**) The percent relative weight change was monitored daily over 7 days. Each group (*n =* 3) was challenged intranasally with 10^5^ TCID_50_ of SARS-CoV-2 and intraperitoneally injected with different doses of humanized Abs or control treatments (isotype and PBS controls) at 3 hours and again at 1 day after infection. Significance was determined by 2-way ANOVA followed by Tukey multiple-comparison test. (**B**) The lung viral titers were measured by TCID_50_ assay at 3 dpi. (**C**) Representative images of lung cross-sections depict changes in pathology at 6 dpi. H&E-stained sections are shown. (**D**) Lung injury scores were assessed on the basis of the percentage of inflammation area in each section of each animal. (**B** and **D**) The data were statistically analyzed by ordinary 1-way ANOVA followed by Tukey multiple-comparison test. All data are reported as the mean ± SEM. *P < 0.05, **P < 0.01, ****P < 0.0001 compared with the control.

Additionally, we further purified the candidate mAbs and the chimeric counterpart from expressed supernatants to determine an accurate binding constant and IC_50_ values for subsequent identification of the mAb with the best antigen-binding affinity and neutralizing capability against SARS-CoV-2 (Supplemental Table 2 and Figure 4). Thus, we chose the top humanized mAb, hMab5.17, for further investigation because it displayed favorable affinity, with a *K_D_* of 13 pM, and potent neutralizing ability, with an IC_50_ value of 12.2 μg/mL, similar to its parental Mab5. Of note, hMab5.17 had a strikingly slower off-rate constant in binding with the S2 protein (10^–6^/s), which indicated that it has strong antigen-binding ability (Supplemental Figure 4A and Supplemental Table 2).

Using different densities of antigen loaded onto BLI sensors to assess the optimal loading molecule concentration, we ruled out the possibility of avidity-based interaction (Supplemental Figure 4B). All BLI experiments in this study were performed using the optimal concentration of 500 nM for antigen loading because no saturating loading was observed for the bound Ab under this condition, which would allow comparison of the *K_D_* values of all Abs and resulted in fixing the response to 1.0 nm.

### Protective efficacy of humanized hMab5.17 against SARS-CoV-2 challenge in Syrian hamsters.

To further validate the in vivo potency of humanized hMab5.17, we evaluated the dose-dependent protective efficacy through the passive administration of neutralizing Abs in the Syrian hamster model. Thirty hamsters were divided into 5 groups of 6 animals each and received an intraperitoneal injection of 10 mg, 5 mg, or 2.5 mg of hMab5.17 per animal, compared with 10 mg of isotype control or PBS alone. Animals were treated for 3 hours and again at 1 day after infection, and parameters were determined as described in Figure 3.

As expected, when examined 6 days after virus challenge, the animals that had been administered 10 mg or 5 mg of hMab5.17 had no weight loss, whereas a slight weight loss was detected at 5 days postinoculation (dpi) in the animals administered 2.5 mg of the Ab (Figure 4A). In contrast, animals in the isotype control-treated and PBS control groups did not recover their initial body weight by 6 dpi. In terms of the reduction in viral replication and pathology, the 3 doses significantly led to a reduction in viral replication (TCID_50_) at 3 dpi (Figure 4B), and an analysis of the lung pathology showed minimal to mild pathological changes in all hMab5.17-treated hamsters at 6 dpi (Figure 4, C and D). The isotype control groups had severe lesions upon histopathological analysis, consistent with the cumulative lung histopathological score. Therefore, the in vivo and in vitro evidence revealed that humanized hMab5.17 successfully retained mouse parental Ab specificity and therapeutic efficacy against SARS-CoV-2 infection in the Syrian hamster model.

### Neutralization activity and protective efficacy of hMab5.17 against SARS-CoV-2 variants.

**Figure 5:**
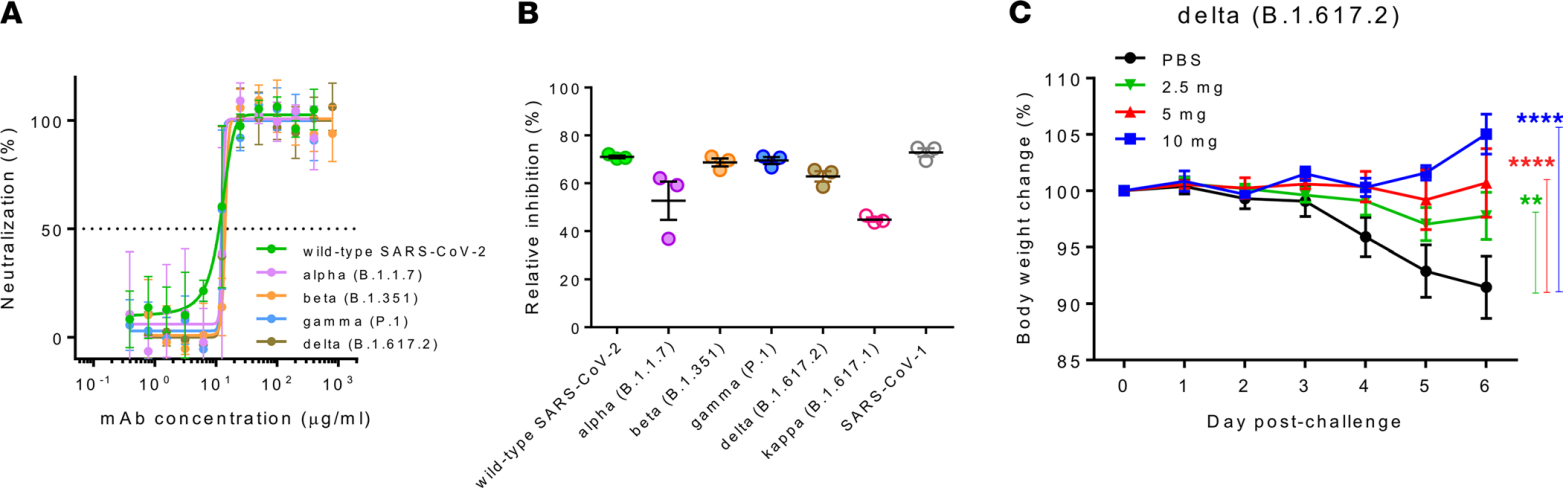
Neutralization by hMab5.17 targeting WT and variant SARS-CoV-2. (**A**) Neutralizing activity of hMab5.17 against the authentic Alpha, Beta, Gamma, and Delta viruses compared with that against the original SARS-CoV-2 strain. The horizontal dotted line indicates 50% neutralization. (**B**) hMab5.17 neutralized the pseudoviruses SARS-CoV-2, SARS-CoV-1, and some dominant SARS-CoV-2 S natural variants. Restriction of pseudovirus entry by humanized Abs is shown as a percentage of relative inhibition. The filled circles show the corresponding authentic viruses in **A**, and empty circles indicate only pseudotyped viruses. (**C**) hMab5.17 showed high therapeutic efficacy in Delta variant–infected hamsters. The body weight change (%) was monitored daily over 6 days. Each group (*n =* 4) was challenged intranasally with 10^3^ TCID_50_ of the SARS-CoV-2 Delta variant and intraperitoneally injected with different doses of humanized Abs or PBS control treatments 3 hours and again at 1 day after infection. Significance was determined by 2-way ANOVA followed by Tukey multiple-comparison test. The data are reported as mean values. All data are reported as the mean ± SEM. **P < 0.01, ****P < 0.0001 compared with the control.

Because SARS-CoV-2 VOCs harbor multiple mutations in the S1 subunit rather than the more conserved S2 subunit to confer immune escape from neutralizing Abs (35), we next estimated whether hMab5.17 targeting the HR2 domain on the S2 subunit could be effective against currently spreading mutants. To address this question, we performed a live-virus CPE neutralization assay in parallel with a lentivirus-based pseudovirus assay with pseudoviruses bearing the indicated spike mutations (36). Surprisingly, hMab5.17 displayed very uniform neutralizing titers against the UK-dominant Alpha variant (B.1.1.7), Brazil-dominant Gamma variant (P.1), South Africa–dominant Beta variant (B.1.351), and the Delta variant (B.1.617.2) that spread throughout India, similar to its activity against WT SARS-CoV-2, and the IC_50_ value was approximately 12 μg/mL (Figure 5A and Supplemental Figure 5C). As expected, in agreement with the live-virus assay results, hMab5.17 exhibited corresponding inhibitory activity against infection by pseudotyped variants as well as by pseudotyped SARS-CoV-1 (Figure 5B). Notably, although V^1176^ to F^1176^ was the only mutated residue in the highly conserved HR2 domain of the Gamma (P.1) strain, this mutation did not affect the neutralizing activity of hMab5.17 (Figure 5, A and B, and Supplemental Figure 5A).

For assessment of the in vivo protection in animals against variant infection, we challenged hamsters with the Delta variant and found that treatment with 10 mg and 5 mg of hMab5.17 resulted in significant prevention of weight loss, whereas mild weight loss was detected in the group administered 2.5 mg of the Ab (Figure 5C). hMab5.17 exhibited potent therapeutic effectiveness in Delta SARS-CoV-2 variant–infected hamsters, which indicated that the protective ability of the Ab in animals against the Delta strain was comparable to that in hamsters infected with the WT strain (Figure 4) that were administered the same doses of the Ab. Collectively, these results revealed that humanized hMAb5.17 targeting the stem-helix epitope in the S2 subunit conferred broad neutralization in vitro against infection by SARS-CoV-1, SARS-CoV-2, and emerging SARS-CoV-2 VOCs and showed substantial in vivo protection in Delta variant–infected hamsters.

### Structure of the fusion core and identification of the critical amino acid residues on the CB-119 epitope.

**Figure 6:**
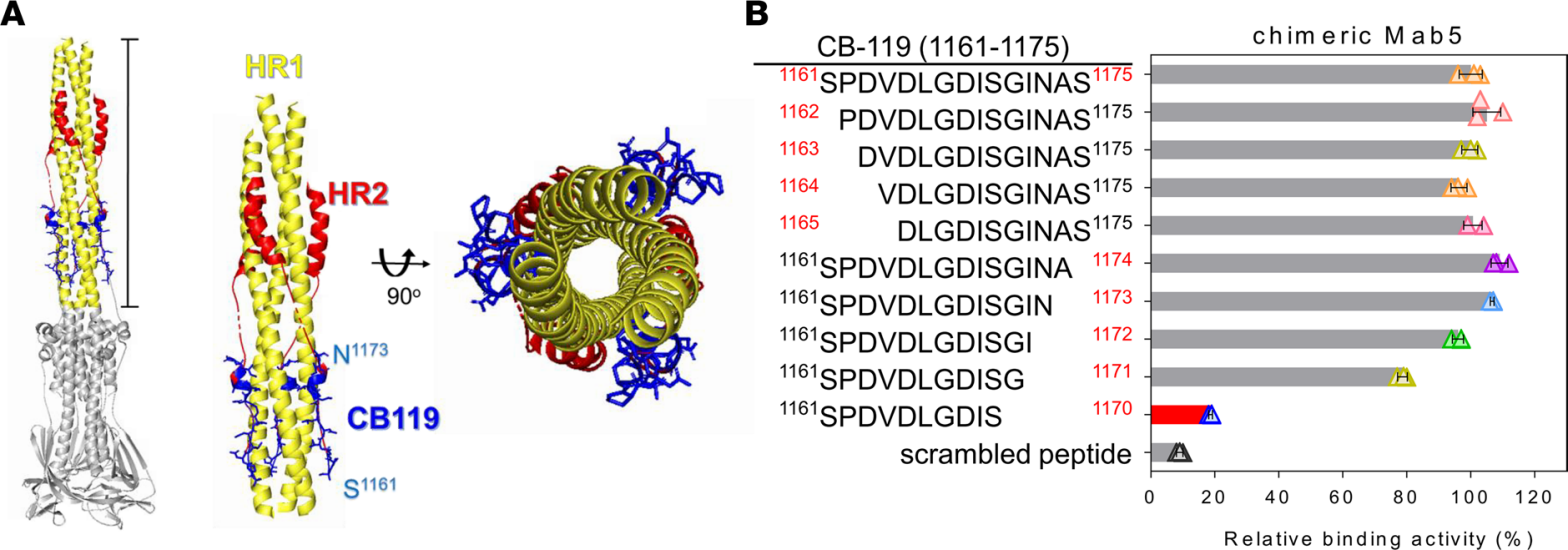
Structure of the fusion core and identification of the critical amino acid residues on the CB-119 epitope. (**A**) Overall views of the SARS-CoV-2 S2 trimer in the postfusion machinery (left) and locations of the CB-119 epitopes (blue) in the S2 structure (Protein Data Bank: 6XRA). The zoomed-in views show the fusion core from the side view (middle), and the second view (right) has been rotated 90° to show the exposed CB-119 epitope on the surface of 3 HR1 helices. Various structural components are represented by the following color scheme: HR1 (yellow), HR2 (red), CB-119 (blue), and the N-/C-terminal residues of CB-119 (cyan). (**B**) Identification of the minimal Mab5 epitope was performed by ELISA. Various N- and C-terminal truncated peptides were synthesized to detect reactivity with chimeric Mab5 and labeled in the left panel with the residue numbers of the amino acids. The percentage of relative binding activity is displayed and was normalized to the binding activity level of the CB-119 peptide. The red bar indicates the weakest reaction intensity among the synthetic peptides. The data denote the means ± SDs from experiments performed independently at least 3 times.

According to knowledge of coronavirus infection, the formation of a fusion core from the native prefusion conformation undergoing a transition state into a stable postfusion is mediated by HR1 and HR2 of the S2 subunit and results in a stable tight structure called a 6-helix bundle fusion core (37, 38). Mab5 specifically recognized a site located in the HR2 region, which is adjacent to the transmembrane domain and might cause steric hindrance of mAbs. To investigate whether the neutralizing epitope CB-119 is exposed and accessible for Mab5 binding, we positioned CB-119 in the crystal structure of the S2 fusion core in the postfusion hairpin conformation (Figure 6A). Notably, because the S stem helix in many prefusion structures is not fully resolved (26, 27), only high-resolution information of postfusion structures is available for docking the position of CB-119 (38). In fact, ChiMab5 can bind to the S2 subunit in both the prefusion and postfusion states with comparable avidity to the CB-119 peptide, as determined by ELISA, but not to the S1 and RBD (Figure 1, C and D, and Supplemental Figure 2D). Therefore, we infer that CB-119 may be well exposed in both conformational states for Mab5 binding to interfere with final fusion complex formation.

Inspired by this available mapping structure (Figure 6A), CB-119 was first folded into an extended loop along with a 1-turn helical conformation (Supplemental Figure 5B). On the other hand, CB-119, belonging to the HR2 extended N-terminal loop, has surface exposure because it wraps around the central triple helical bundle of HR1 (Figure 6A, right panel) but is relatively flexible, resulting in 2 residues of C-terminal CB-119 (A^1174^ and S^1175^) remaining unresolved. Accordingly, we further defined the critical contact residues with Mab5 as determined by ELISA with synthetic truncated peptides. Notably, the minimal core epitope D^1165^LGDISGIN^1173^, folding to form 1 turn of an α helix, was essential for binding (Figure 6B and Supplemental Figure 5B). Collectively, our findings elucidated that Mab5 might target well-exposed CB-119 in both conformational states and that residues from D^1165^ to N^1173^ are the only distinct 3D architecture of CB-119 available for Ab binding. However, structural analysis is still needed for a detailed understanding of how Mab5 binds to the minimal epitope, resulting in neutralization of SARS-CoV-2 infection.

## Discussion

In summary, our study characterized 2 murine anti-SARS-CoV-1 mAbs targeting a highly conserved and immunogenic epitope in the membrane-proximal stem of the HR2 region on the S2 subunit. Both Abs not only cross-reacted with SARS-CoV-2 (Figure 1) but also efficiently inhibited SARS-CoV-2 infection in vivo in Syrian hamsters (Figure 3). Furthermore, we humanized a promising candidate mAb, Mab5, which exhibits potent neutralizing activities in vitro and therapeutic effects in vivo similar to those of the parental mouse Ab (Figure 4). Here, we describe a SARS-CoV-2–specific cross-neutralizing mAb targeting the highly conserved S2 subunit and further demonstrate its broad neutralizing capacity and substantial in vivo protection against SARS-CoV-2 variants. These findings provide an opportunity for the development of universal vaccines and Ab-based therapies against current pandemic strains as well as future SARS-CoV-2 mutants.

To date, S2-specific mAbs against SARS-CoV-2 have rarely been reported. Only a few anti-S2 mAbs with neutralizing potency or cross-reactivity to SARS-CoV-2 have been reported (24–31); these are listed in Table 1. These reported S2-specific mAbs isolated after heterologous coronavirus S protein immunization in mice or from COVID-19 convalescent serum all had cross-reactivity with SARS-CoV-1 and/or other human-infecting coronaviruses. Our anti-S2 mAbs, Mab5 and Mab3-2, which were isolated from mice immunized with SARS-CoV-1 S protein (32), recognized the neutralizing epitope (CB-119) located at the N-terminal end of the HR2 domain, which displays 100% identity across strains (Figure 1A and Supplemental Figure 1A). Through direct comparison of the individual epitopes targeted by 6 other reported anti-S2 mAbs (Supplemental Table 1), we surprisingly found that they recognized a highly conserved region in the stem helix of the SARS-CoV-2 S2 sequence K^1149^EELDKYFKN^1158^ (for CC40.8, CV3-25, and S2P6) that corresponded to the Middle East respiratory syndrome–related coronavirus sequence D^1233^ELDEFFKN^1241^ (for B6, 28D9, and 1.6D7), which resulted in conferring the greatest cross-reactivity and neutralization breadth to human-infecting betacoronaviruses (Table 1) (24–31). However, our cross-reactive mAbs recognized regions downstream of their epitopes with the partially overlapping sequence S^1161^PDVDLG^1167^ (for CC40.8 and CV3-25), which was only conserved in SARS-CoV–like viruses, resulting in restricted cross-reactive responses but no responses to other related coronaviruses (Figure 1B) (24, 26, 30). Although our isolated mAbs recognized different residues and the stem-helix-targeting Abs recognized a similar epitope near the HR2 region, they all effectively blocked virus infection in animals and thus induced prophylactic and therapeutic protection (Table 1). Collectively, the SARS-CoV-2 S2 subunit, particularly the N-terminal end of HR2, is highly immunogenic and efficient in eliciting the production of broad-spectrum neutralizing Abs, which suggests the possibility of pancoronavirus intervention (20, 21).

It is worth mentioning that although neutralization was strongly associated with therapeutic protection in this study, many studies have shown that the constant (Fc) region of Abs can also contribute to protective efficacy in vivo to block viral entry and enhance clearance of infected cells (28, 39, 40). This evidence, as previously described for some SARS-CoV-2 cross-reactive mAbs, such as S2P6 (28), CV3-25 (25), S309 (41), and CR3022 (42), indicates that Fc-mediated effector functions may offer auxiliary mechanisms of protection in animals against infections. From this point of view, more investigation is needed to elucidate the exact role of Mab5-mediated coronavirus neutralization associated with Fc-mediated functions that contribute to the prevention of SARS-CoV-2. The inferred mechanism of Mab5 will have an important impact on the design of Ab therapeutics.

Additionally, some structural analysis of the trimeric betacoronavirus S ectodomain in complex with an anti-S2 neutralizing Ab revealed that both prefusion and postfusion spikes can be bound by these known Abs, such as CC40.8, CV3-25, S2P6, and B6. Although the entire S2 subunit structures of both conformations with highly flexible stalk regions are not fully resolved, based on the epitope overlapping with CC40.8 and CV3-25, mentioned above, as well as studies of their structures (24, 26, 30), we speculate that Mab5 can target both conformational states because of the well-exposed CB-119, which is also in line with our ELISA-binding results (Figure 1D and Supplemental Figure 2D) and epitope mapping predictions (Figure 6A). Thus, a putative mechanism of Mab5 neutralization might involve steric interference with the fusion machinery to block viral entry during SARS-CoV-2 infection of Vero cells (Figure 2C and Figure 5A). However, we cannot rule out the possibility that Mab5-mediated neutralizing activity might be blocked through a yet-unknown mechanism different from binding interference, because a plurality of options allow SARS-CoV-2 entry during infection (43, 44), such as the sequential endocytosis of SARS-CoV-2 infection observed in Vero E6 cells (45). The viral-Ab complex may prevent infections by interfering with virus binding to receptors, blocking uptake into cells, preventing uncoating of the genomes in endosomes, or causing virion aggregation (46). Most importantly, we determined that Mab5, indeed, played a protective role in mitigating viral infection in live animal experiments (Figures 3–5), which makes our identified Ab a promising compound for therapeutic strategies.

Another attractive approach to interfere with viral infection, apart from mAb blockade, is using inhibitory drugs or peptides targeting domains of the membrane fusion architecture (47). Peptides derived from the relatively conserved epitopes in the HR regions of coronaviruses have been proven capable of effectively inhibiting viral-cell membrane fusion to prevent viral infection (23, 48). Many studies have revealed synthetic peptides derived from the HR2 region, including partial or complete residues of CB-119, with remarkable inhibitory activity against coronavirus infection (49, 50). Moreover, authors of another study revealed that the linear epitope S2-47, which partially overlaps with CB-119, impaired the neutralizing activity of COVID-19 convalescent sera (51). On the basis of knowledge from the aforementioned studies, it is apparent that the S2 subunit may be a better antiviral target for the development of broad-spectrum prophylactic or therapeutic agents.

Because S2 is markedly more conserved and less prone to retain mutations than is S1, we analyzed and demonstrated the ability of hMab5.17 to neutralize emerging natural variants, including Alpha (B.1.1.7), Beta (B.1.351), Gamma (P.1), Delta (B.1.617.1), and Kappa (B.1.617.1) (Figure 5 and Supplemental Figure 5C). However, only 2 reported anti-S2 mAbs (CV3-25 and S2P6) isolated from patients with COVID-19 displayed a neutralizing response against variants (Table 1) (24–26, 28). As demonstrated in vivo through animal protection experiments, hMab5.17 exhibited potent therapeutic effectiveness in hamsters infected with the Delta (B.1.617.1) variant of SARS-CoV-2 (Figure 5C), which was in line with the finding that CV3-25 exhibits in vivo protection against both Alpha (B.1.1.7) and Beta (B.1.351) variants in the K18-hACE2 prophylactic mouse model (25, 26).

At the beginning of November 2021, the newly emerged omicron variant belonging to Pango lineage B.1.1.529 was found to display an unusually large number of mutations in the S protein, which is of grave concern (52). Excitingly, the neutralizing epitope CB-119 on the S protein of omicron (B.1.1.529) had no mutation. Therefore, our study highlights that Abs whose production is elicited by CB-119 appear to target a potential Achilles’ heel of SARS-CoV-2, especially variants arising as the pandemic spreads, by interference with membrane fusion to arrest the entry of viruses. The rational development of mAb therapies in the future through the combination of 2 or more mAbs targeting RBD epitopes or conserved non-RBD regions as Ab cocktails may have more advantages for therapeutic applications in pan–SARS-CoV protection (53, 54).

## Methods

### Cell lines.

Human embryonic kidney HEK293T and Vero cells (ATCC) were incubated at 37°C in the presence of 5% CO_2_. HEK293T cells were maintained in DMEM (Gibco) containing 10% FBS (HyClone), 100 U/mL penicillin/streptomycin (Gibco), and 2 mM l-glutamine (Gibco). Vero cells were cultured in M199 medium (Gibco) supplemented with 5% FBS. ExpiCHO cells (Thermo Fisher Scientific, A29127) were maintained according to the manufacturer’s instructions at 37 °C in 8% CO_2_ in ExpiCHO Expression Medium (Thermo Fisher Scientific, A2910002).

### ELISA quantification.

ELISA plates were coated with 50 μL/well of 2 μg/mL protein and 20 μg/mL peptides in 0.1 M carbonate buffer (pH 9.5) at 4°C overnight. Antigen was removed by standard washing, and the plates were blocked with 200 μL of 3% BSA (Sigma, A9647) for 2 hours at room temperature. The neutralizing Abs were serially diluted using 1% BSA in PBS and incubated for another 2 hours at room temperature. The binding signal was detected using HRP-conjugated goat anti–human IgG (ARG23887, Arigo) or anti–mouse IgG (31436, Invitrogen) and visualized by 3,3′, 5,5′ tetramethylbenzidine dihydrochloride substrate (BioLegend, 421101). After the reaction was stopped by the addition of 1 M sulfuric acid, the absorbance was measured with an ELISA reader at 450 nm.

### Ab expression and purification.

For chimeric and humanized Abs, all sequences were codon optimized for human expression and synthesized by GenScript Corporation, which also offers custom-designed back-mutations in humanized murine Abs and ranks the binding kinetics using a Biacore 8K. The variable regions were cloned separately into a pcDNA3.4 expression cassette containing cattle IgG constant region-coding sequences. The recombinant mAbs were expressed in ExpiCHO cells (Thermo Fisher Scientific, A29127) following the instructions of the manufacturer. Ab-containing supernatants were collected 14 days after transfection and purified by Protein A-Sepharose (GE, 17-1279-01) affinity chromatography.

### IFA.

Vero cells were seeded in a 24-well plate and infected with SARS-CoV-2 (MOI = 0.1). After 24 hours, the cultures were fixed in 4% paraformaldehyde in PBS at room temperature for 30 minutes and permeabilized with 0.1% Triton X-100 (Sigma) in PBS for 15 minutes. The cells were then washed 3 times with PBS and blocked with 1% BSA–PBS blocking solution for 30 minutes. Immunostaining was performed using purified Abs at 1:1000 dilution in blocking solution and incubated for 60 minutes at room temperature. After the primary Ab was removed by washing with PBS, visualization was performed through incubation with FITC-conjugated goat anti–mouse IgG secondary Abs (Invitrogen, Thermo Fisher Scientific; F-2761, dilution 1:5000) for 60 minutes at room temperature. The stained cells were washed with PBS, and the nuclei were then counterstained with 1 μg/mL nuclear DAPI (Sigma, D9542). Fluorescent images were obtained with a fluorescence microscope (Olympus IX73).

### TCID_50_ assays.

For the neutralization assay, authentic WT SARS-CoV-2 (hCoV-19/Taiwan/4/2020) and variants (alpha-hCoV-19/Taiwan/792/2020, beta-hCoV-19/Taiwan/1013/2021, gamma-hCoV-19/Taiwan/906/2021, and delta-hCoV-19/Taiwan/1144/2021) were obtained from the Centers for Disease Control in Taiwan. The viral titer causing CPE was estimated using the TCID_50_ determined via a standard method (55). Briefly, 2-fold serial dilutions of each neutralizing Ab were prepared in M199 medium. Thereafter, 200 TCID_50_ of SARS-CoV-2 was mixed in a 1:1 (vol/vol) ratio. After 2 hours of incubation at 37°C, the mixture was inoculated onto a Vero cell monolayer (2.4 × 10^4^ cells/well) in 96-well plates in quadruplicate and incubated at 37°C. The CPE was scored after 4 days of infection. The neutralizing Ab titer was interpreted as the highest dilution with 50% of the CPE in the inoculated wells. All assays with viruses were conducted in a biosafety level 3 laboratory.

### SARS-CoV-2 neutralization assay.

After CPE observation, the cells were fixed by immersion in 10% formaldehyde in PBS for 24 hours. The fixative was removed, and the cells were stained with 50 μL/well 0.5% crystal violet for 20 minutes at room temperature (56). After rinsing the plates with water and drying, 200 μL/well methanol was added to dissolve the crystal violet prior to optical density determination at 570 nm. The IC_50_ values of the neutralizing percentage were calculated using GraphPad Prism, version 7.0, by fitting the data to a 4-parameter nonlinear regression.

### Binding-affinity determination using BLI.

A ForteBio Octet RED BLI system (Octet RED, ForteBio) was used to analyze the binding kinetics of the purified Abs to recombinant His-tag–fused S2 (amino acid residues from 1047 to 1210) protein. The BLI assay buffer consisted of 0.05% Tween 20 (Sigma) in PBS (PBST), which was filtered through a 0.22-μm filter. Recombinant S2 proteins at a concentration of 10 μg/mL (500 nM) were immobilized on an Anti–Penta-HIS (HIS1K, ForteBio, 18-5120) biosensor for 100 seconds, and a baseline reading was recorded for 30 seconds in PBST. The loaded tips were then immersed into wells containing different concentrations of mAbs, allowed to associate for 250 seconds, and then dissociated for 300 seconds. All assay steps were performed at 24°C with agitation at 1000 rpm. Kinetic values were calculated by ForteBio data analysis software using a 1:1 binding model in Data Analysis Software, version 8.2.

### Animal challenge.

The Syrian hamster model was established for the in vivo evaluation of SARS-CoV-2 neutralization in accordance with previously reported guidelines (57). Each female hamster (aged 8 to 10 weeks, approximately 150 g; purchased from the National Laboratory Animal Center) was intranasally inoculated with a total volume of 50 μL containing a dose equal to 10^5^ TCID_50_ of WT SARS-CoV-2 or 10^3^ TCID_50_ of the Delta variant in PBS. The lower 10^3^ TCID_50_ of the Delta variant had the same toxicity as the WT, as demonstrated by a 10% weight loss in the hamsters.

To determine the therapeutic efficacy of murine mAbs, each hamster (*n =* 10) was treated with 2.5 mg (16.5 mg/kg) of Mab5 or Mab3-2 or PBS alone as a control via intraperitoneal injection at 2 hours and again at 24 hours after infection. For determination of the therapeutic efficacy of the humanized mAb (*n =* 6 or *n =* 8), each animal was intraperitoneally injected with 10 mg (66.6 mg/kg), 5 mg (33.3 mg/kg), or 2.5 mg (16.5 mg/kg) of hMab5.17 or 10 mg of the isotype control or PBS alone at 3 hours and again at 24 hours after infection. The body weight was monitored daily for at least 6 continuous dpi. The hamsters in each group were sacrificed at 3 dpi, and the lungs were harvested for virological and histopathological analyses. The remaining animals were sacrificed at 6–11 dpi. All animals were housed at the Animal Center of the NHRI and maintained in accordance with institutional animal care protocols.

### Quantification of viral load.

Left-side lung tissues were homogenized in 2 mL of PBS using a gentleMACS Dissociator (Miltenyi Biotec) according to the manufacturer’s protocol. Tissue homogenates were used for infectious virus titration by TCID_50_ assay and viral load determination by reverse transcription–quantitative PCR (RT-qPCR) assay. RNA was extracted with TRIzol LS (Ambion) according to the manufacturer’s instructions. Ten nanograms of the extracted RNA was subjected to real-time RT-qPCR performed on a QuantStudio 6 Flex Real-Time PCR System (ABI) using a KAPA PROBE FAST Universal One-Step RT-qPCR Kit (KR1282, Roche) with primers and probes specific for SARS-CoV-2 (E_Sarbeco forward: ACAGGTACGTTAATAGTTAATAGCGT, E_Sarbeco reverse: ATATTGCAGCAGTACGCACACA, E_Sarbeco probe: FAM-ACACTAGCCATCCTTACTGCGCTTCG-BHQ1) (58).

### Histological analysis and pathologic score.

Lungs were harvested and fixed in 10% phosphate-buffered formalin and embedded in paraffin using routine methods. Sequential sections were stained with H&E to assess pathology and lung damage. Each histologic section was also used for analysis of general lung pathogenic lesions by the Pathology Core Laboratory (NHRI). Each histopathologic change in a section of total lobes was scored on the basis of histopathologic parameters such as peribronchiolitis, perivasculitis, interstitial pneumonia, and alveolitis on a scale of 0 (no change) to 4 (maximum inflammation) by a clinical pathologist.

### Pseudotyped virus neutralization assay.

The neutralization activity was analyzed according to the description in a previous study (36). Briefly, hACE2-expressing BHK-21 (BHK-hACE2) cells were seeded in 24-well plates and incubated at 37°C for 24 hours. The pseudovirus was incubated with 10 ng/μL hMab5.17 in a final volume of 200 μL/well with serum-free medium at 37°C for 1 hour and then added to BHK-hACE2 cells for another 6 hours. After washing the cells twice with PBS, the complete cell-culture medium was replaced. Total cell lysates were harvested at 48 hours after infection, and pseudovirus entry was analyzed by determining the luciferase activity. The relative inhibition of pseudovirus infectivity was calculated relative to the control group.

### Epitope mapping to the structure.

To visualize the position of the mAb binding site, the CB-119 epitope was mapped in the SARS-CoV-2 S2 trimer structure (Protein Data Bank: 6XRA) in postfusion machinery and manually adjusted using the educational version of PyMOL software.

### Statistics.

The statistical data were analyzed and plotted using GraphPad Prism, version 7.0. The statistical significance was evaluated by 1-tailed Student *t* test, 1-way ANOVA followed by Tukey multiple-comparison test, or 2-way ANOVA followed by Tukey multiple comparison test. *P* < 0.05 was considered statistically significant.

### Study approval.

All animal experimental protocols were approved by the IACUC at the NHRI (protocol no: NHRI-IACUC-109077-A).

## Author contributions

WLW, SJL, and HWC contributed to the conception and design of the experiments. WLW, CYC, SCL, YLH, HCL, and CYY performed the experiments. All authors contributed to the analysis and interpretation of the results. CLL, WLW, and SJL led the manuscript writing. All authors participated in the manuscript writing, editing, and critical revision.

## Supplementary Material

Supplemental data

## Figures and Tables

**Table 1:**
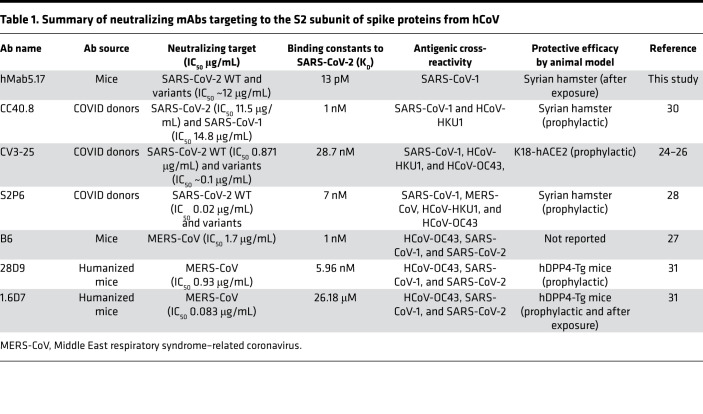
Summary of neutralizing mAbs targeting to the S2 subunit of spike proteins from hCoV

## Version 1 03/15/2022

In-Press Preview

## Version 2 04/22/2022

Electronic publication
