# Supplementary material for: Monoclonal antibody targeting the conserved region of the SARS-CoV-2 spike protein to overcome viral variants
Source: JCI Insight. 2022 Apr 22;7(8):e157597. doi: 10.1172/jci.insight.157597 (PMC9089791; doi:10.1172/jci.insight.157597)
Supplement: Supplemental data [file jciinsight-7-157597-s153.pdf]

Supplemental data

Monoclonal antibody targeting the conserved region of the SARS-CoV-2 spike protein to overcome viral variants

Wan-Ling Wu[1], Chen-Yi Chiang[1], Szu-Chia Lai[2], Chia-Yi Yu[1], Yu-Ling Huang[1], Hung-Chun Liao[1,3], Ching-Len Liao[1], Hsin-Wei Chen[1, 4, 5] and Shih-Jen Liu[1, 4, 5]

[1]National Institute of Infectious Diseases and Vaccinology, National Health Research Institutes, Miaoli, Taiwan
[2]Institute of Preventive Medicine, National Defense Medical Center, New Taipei City, Taiwan
[3]Department of Life Sciences, National Tsing Hua University, Hsinchu, Taiwan
[4]Graduate Institute of Biomedical Sciences, China Medical University, Taichung, Taiwan
[5]Graduate Institute of Medicine, College of Medicine, Kaohsiung Medical University, Kaohsiung, Taiwan

Correspondence to: Shih-Jen Liu and Hsin-Wei Chen
National Institute of Infectious Diseases and Vaccinology, National Health Research Institutes, No. 35 Keyan Road, Zhunan Town, Miaoli County, 350, Taiwan
Tel: 886-3-724-6166 ext. 37709
Fax: 886-3-758-3009
E-mail: levent@nhri.edu.tw

Table of contents:

Supplementary Figure S1

Supplementary Figure S2

Supplementary Figure S3

Supplementary Figure S4

Supplementary Figure S5

Supplementary Table S1

Supplementary Table S2

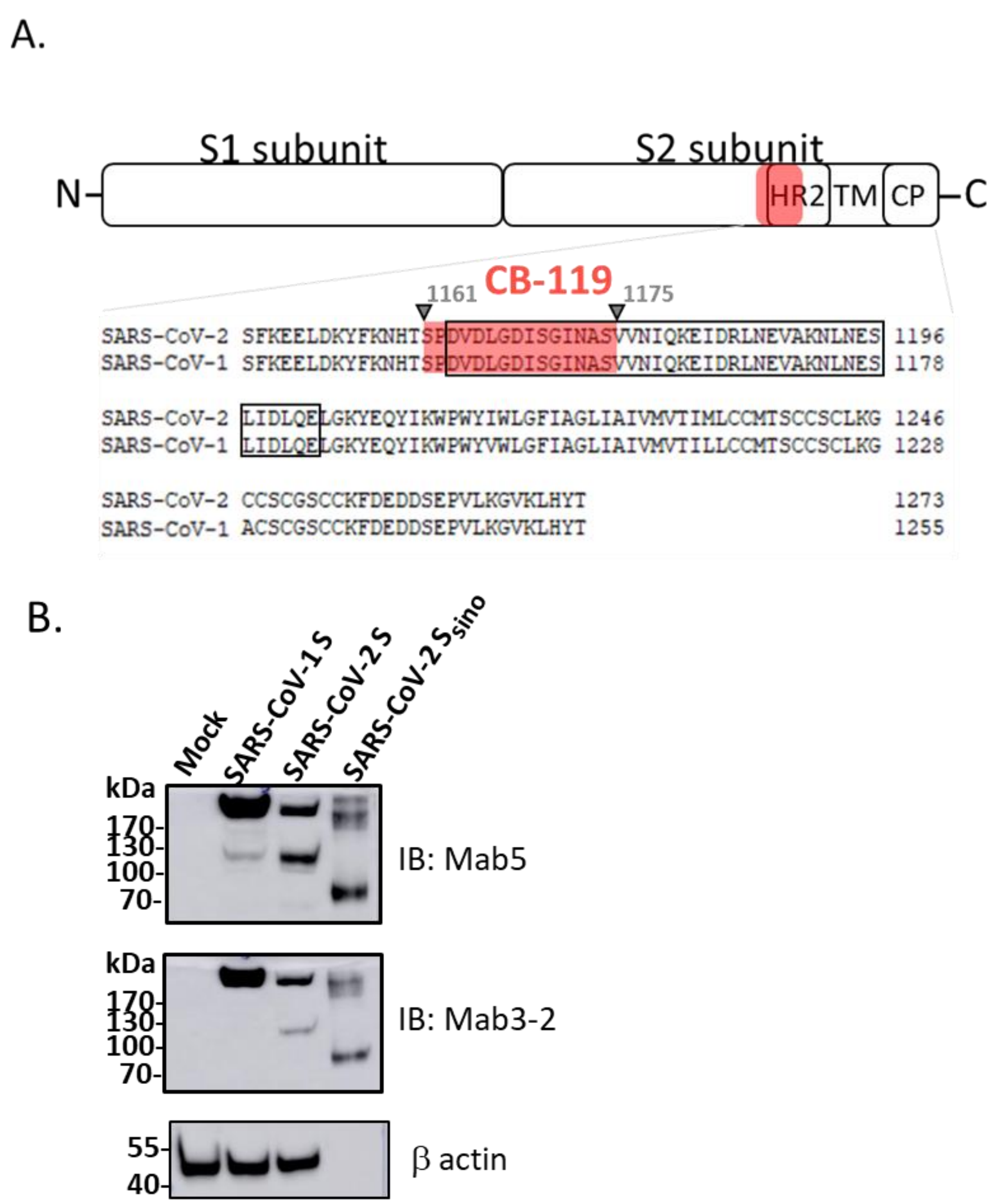

Supplementary Figure S1. Two monoclonal antibodies with cross-species specificity recognize the S2 subunit.

(A) Schematic diagram representing the neutralizing epitope, CB-119, located in the N-terminal HR2 domain within S2 of the SARS-CoV-2 spike protein (S). Sequence alignment of the C-terminal regions from SARS-CoV-1 and SARS-CoV-2 S2 subunits, including the heptad repeat 2 (HR2) domain, transmembrane (TM) domain and cytoplasmic (CP) domain. The boxed residues indicate the HR2 domain and the epitope CB-119 recognized by Mab5 and Mab3-2, which is highlighted with red shading. (B) Expression plasmids encoding SARS-CoV-1 or SARS-CoV-2 S proteins were transiently transfected into 293T cells. Subsequently, Mab5 and Mab3-2 specifically detected the S protein from 293T lysates, as demonstrated by Western blotting. β-actin served as a loading control. SARS-CoV-2 S_{sino} is a recombinant S protein that served as a positive control.

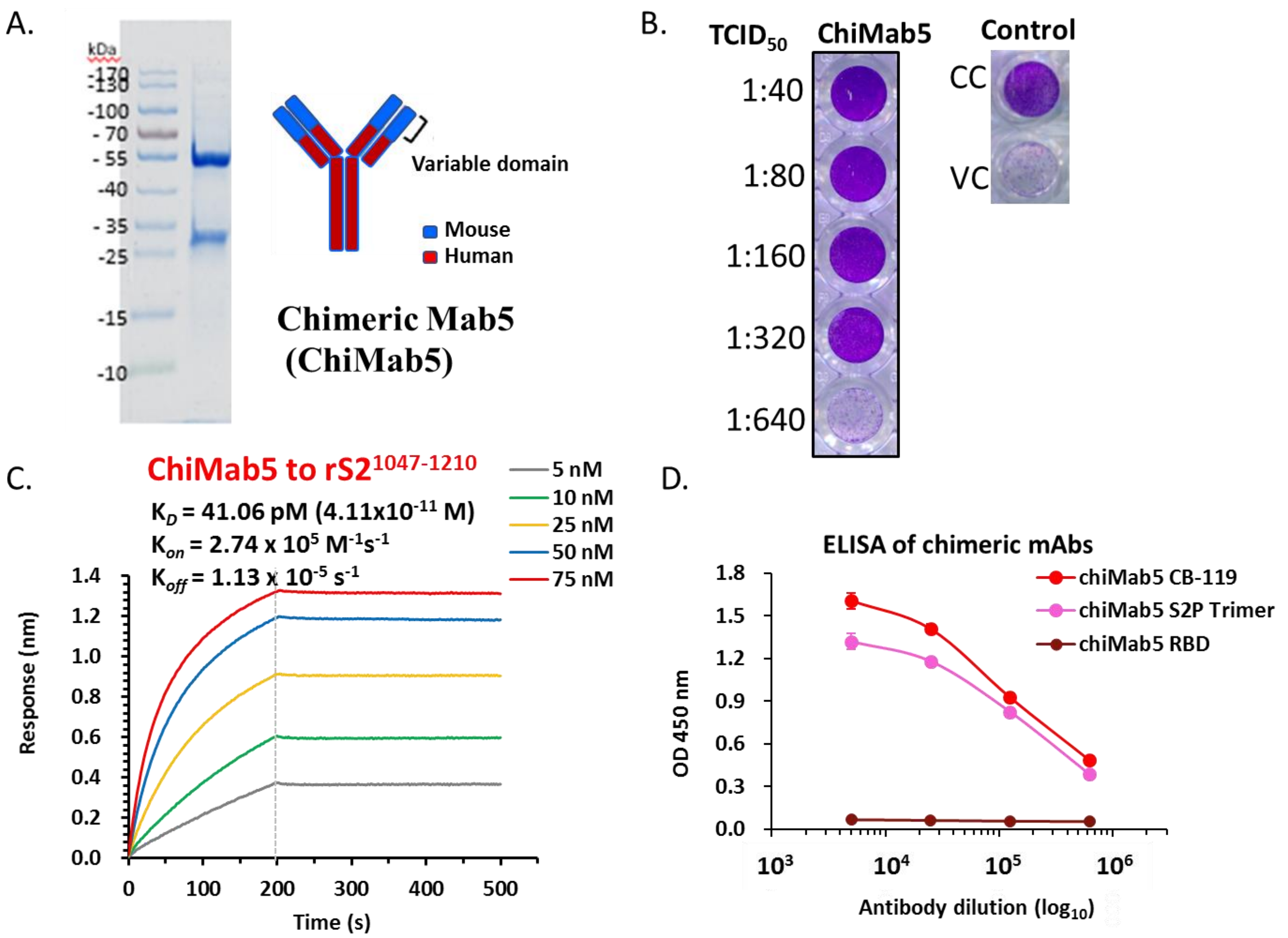

Supplementary Figure S2. Expression, purification and validation of chimeric Mab5 for determination of its neutralization capacity and binding kinetics.

(A) SDS–PAGE analysis of the purified chimeric MAb5. ChiMab5 was expressed in ExpiCHO cells using the standard protocol of the GIBCO ExpiCHO Expression System and purified by Protein A-Sepharose (GE Healthcare Bio-Sciences, Pittsburgh, PA) affinity. The purity of the final preparations was evaluated on Coomassie-stained SDS–PAGE gels. The heavy chain and light chain were approximately 55 kDa and 25 kDa, respectively, on the PAGE gels. The right panel shows a schematic of the ChiMab5 antibody, which combines the constant region of a human antibody with the variable domain of murine Mab5. (B) The neutralization capacity of ChiMab5 was measured by $TCID_{50}$ assay, in which the antibodies were 2-fold serially diluted, and images were visualized by 0.5% crystal violet staining. CC indicates the cell control without virus, and VC indicates the virus control without antibody. (C) The kinetics of ChiMab5 binding to the S2 subunit were evaluated by BLI, which revealed a K_D value of 41.06 pM. One representative of two experiments with similar results is shown. (D) The binding efficacy of the ChiMab5 mAbs for the RBD domain, CB-119 synthesized peptide, and prefusion-stabilized ectodomain trimer S (S2P) was determined by antigen-coating ELISA.

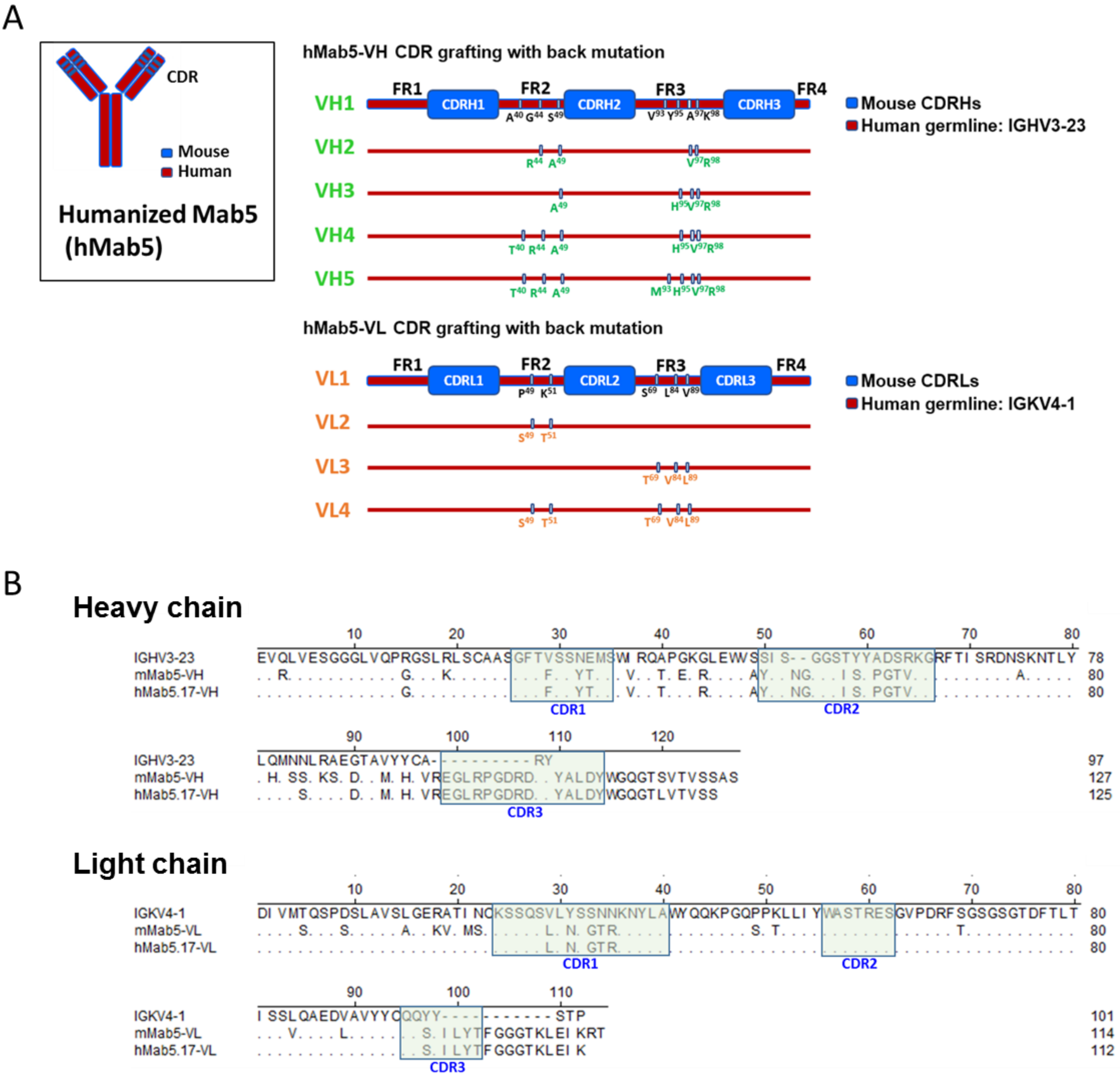

Supplementary Figure S3. Humanization and engineering of chimeric Mab5 by back-mutations onto the human framework templates.

(A) A schematic representation of the humanized Mab5 antibody (right panel), which comprised human germline frameworks (FRs) grafted with murine complementarity determining regions (CDRs) from the Mab5 variable region. The designed back-mutations are highlighted with blocks on the individual human frameworks of the heavy chain or light chain (left panel). Five versions of back-mutated heavy chains (green) and four versions of back-mutated light chains (orange) were arranged and assembled to generate 20 recombinant humanized antibodies. The corresponding antibodies are listed in **Supplementary Table S2**, which also shows the results from further evaluation of their ranking in terms of both binding affinity and neutralizing activity. (B) Alignment of the amino acid sequences of murine and humanized Mab5 with the mature VH and VL (top). The CDR regions of the sequence that correspond to CDR H1, H2, H3, L1, L2, and L3 are indicated and highlighted in shadow boxes.

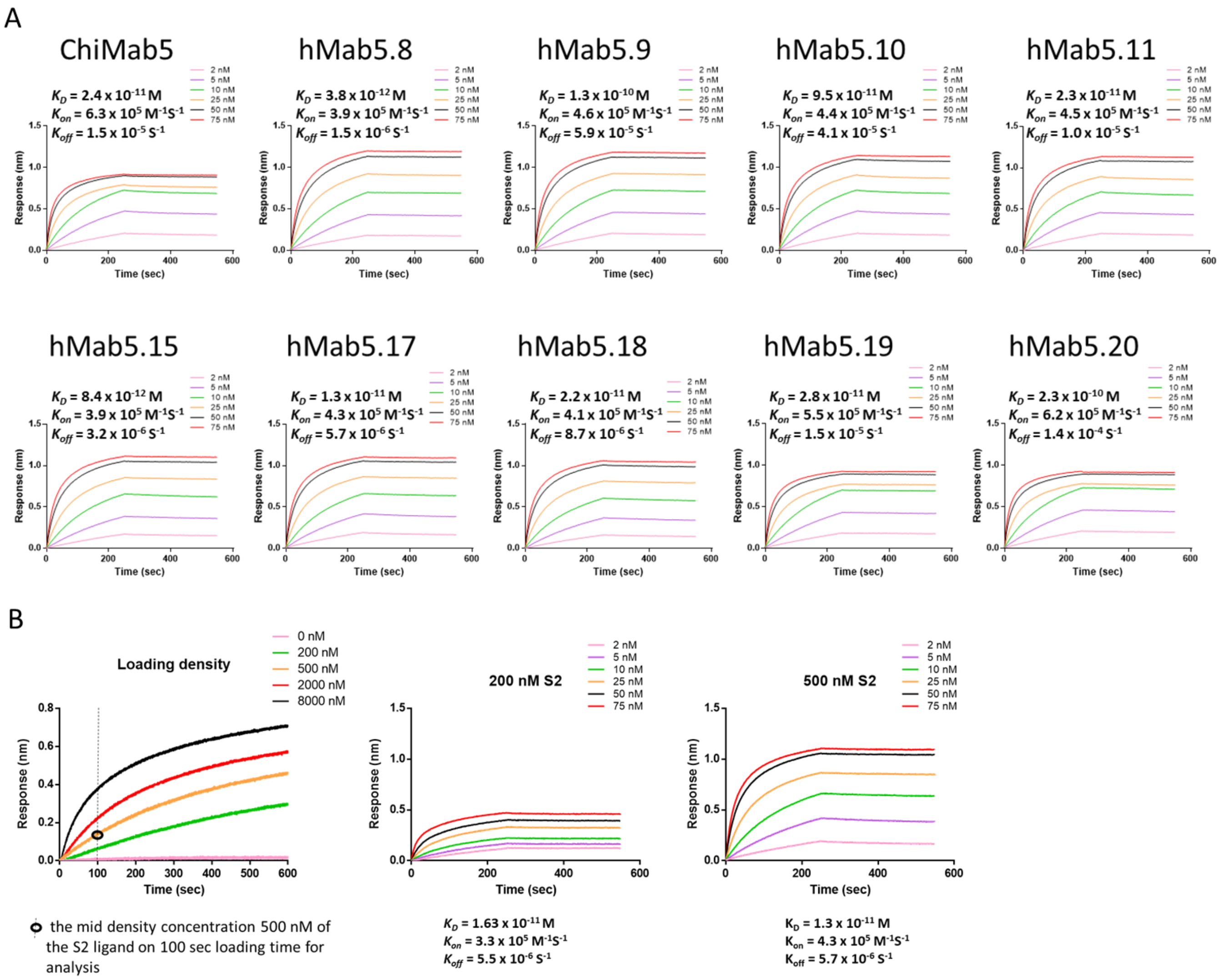

Supplementary Figure S4. Binding kinetics of the chimeric and humanized Mab5 mAbs against the S2 subunit of SARS-CoV-2.

(A) The binding kinetics of chimeric ChiMab5 and nine top humanized anti-S2 hMab5 candidates selected from **Supplementary Table S2** were determined by BLI, which was performed using six different concentrations of mAbs ranging from 2 nM to 75 nM. (B) Antigen loading density scouting and assessment of the optimal loading molecule concentration. The mid density concentration of the antigen loading was 500 nM for 100 s of detection to fix the response at 1.0 nm for all BLI experiments performed in this study.

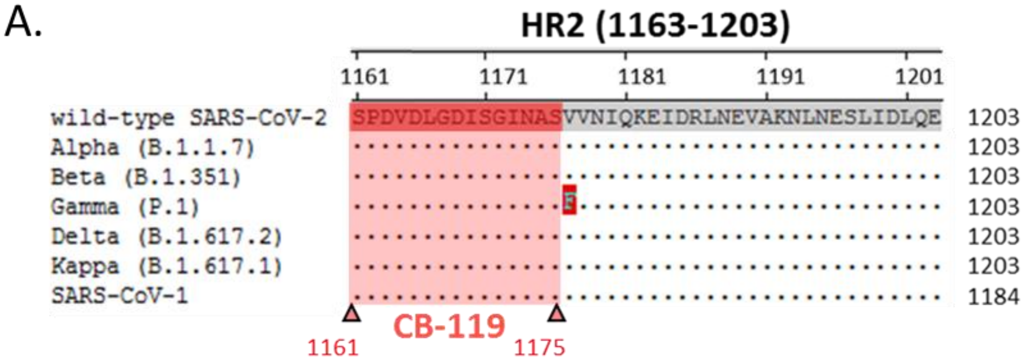

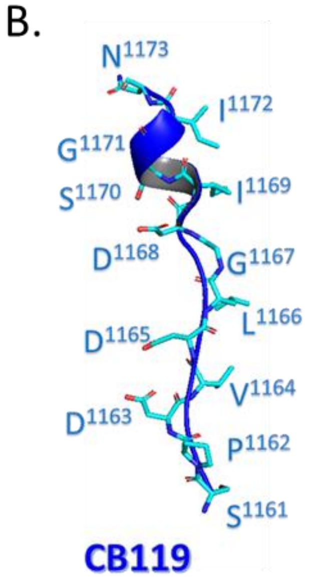

C.

Characterization of hMab5.17 to authentic WT and variants of SARS-CoV-2

mAbs	Authentic virus IC_{50} (μg/mL)
WT	11.2
Alpha	12.9
Beta	14.1
Gamma	12.5
Delta	12.8

Supplementary Figure S5. Sequence and structural properties of the CB-119 epitope in SARS-CoV-2 variants and characterization of the neutralization activity of hMab5.17 against authentic wild-type SARS-CoV-2 and variants.

(A) The sequence alignment of the HR2 regions from SARS-CoV-2 variants is displayed, and the only mutated residue, F^{1176}, in HR2 of the gamma strain is marked in red shading. (B) Cartoon representation of the CB-119 peptide (S1161-N1173) structure in the postfusion state is colored blue, correlating with Figure 6A. The presence of one alpha-helical turn was observed in the CB-119 structure (PDB 6XRA). The amino acid residues in CB-119 are shown by cyan sticks. (C) The neutralizing activity of hMab5.17 targeting authentic wild-type SARS-CoV-2 and four variants, the alpha, beta, gamma, and delta strains, is summarized in Figure 5A with the neutralizing IC_{50} values (50% inhibitory concentrations).

Supplementary Table S1. Immunogenetic properties of the neutralizing monoclonal antibodies targeting the S2 subunit of spike proteins from human coronaviruses.

Antibody name	Epitope target	VH gene	CDR3-H (aa)	VL gene	CDR3-L (aa)	Reference
HMab5	1161-SPDVDLGDISGINAS-1175 $_{SARS\text{-}CoV\text{-}2}$	IGHV3-23*04	EGLRPGDRDRYYALDY	IGKV4-1*01	QQSYILYT	in this study
CC40.8	1140-PLQPELDSFKEELDKYFKNHTSPDV-1164 $_{SARS\text{-}CoV\text{-}2}$	IGHV3-23*01	CAITMAPVVW	IGLV3-10*01	CYSTDSSGNHAVF	(30)
CV3-25	1149-KEELDKYFKNHTSPDVDLG-1167 $_{SARS\text{-}CoV\text{-}2}$	IGHV7-4-1*02	ASARPGVATNLDF	IGKV1-39*01	QQSYSNPLT	(24-26)
S2P6	1146-DSFKEELDKYFKNH-1159 $_{SARS\text{-}CoV\text{-}2}$	IGHV1-46*01	ARGSPKGAFDY	IGKV3-20*01	QQYGSSPPRFT	(28)
B6	1230-DFQDELDEFFKNVST-1244 $_{MERS\text{-}CoV}$	IGHV1-19*01	QLGRGNGLDY	IGKV4-1*01	HQYLSSYT	(27)
28D9	1229-IDFQDELDEFFKNVS-1243 $_{MERS\text{-}CoV}$	IGHV6-1*01	VPMNRGGMDV	IGKV4-1*01	HQYYSIPNT	(31)
1.6D7	1229-IDFQDELDEFFKNVS-1243 $_{MERS\text{-}CoV}$	IGHV6-1*01	ATLARGALDY	IGKV4-1*01	QQYYSTPWT	(31)

Identical residues are indicated by underlined letters.

Supplementary Table S2. Ranking of the binding kinetics and neutralizing abilities of humanized Mab5 mAbs (hMab5).

mAb name	Heavy and light chains	Binding kinetics of expression supernatant[A]			Neutralization[B]	Binding kinetics of purified mAb[C]			IC_{50} (μg/ml)
		K_D (M)	k_{on} ($M^{-1}sec^{-1}$)	k_{off} (sec^{-1})		K_D (M)	k_{on} ($M^{-1}sec^{-1}$)	k_{off} (sec^{-1})	
ChiMab5	chimeric	$4.56x10^{-10}$	$1.14x10^{5}$	$5.18x10^{-5}$	+++	2.4×10^{-11}	6.3×10^{5}	1.5×10^{-5}	15
hMab5.1	VH1+VL1	$1.43x10^{-9}$	$7.14x10^{4}$	$1.02x10^{-4}$	-				
hMab5.2	VH1+VL2	$2.66x10^{-9}$	$5.89x10^{4}$	$1.57x10^{-4}$	-				
hMab5.3	VH1+VL3	$2.40x10^{-9}$	$5.96x10^{4}$	$1.43x10^{-4}$	-				
hMab5.4	VH1+VL4	$2.19x10^{-9}$	$7.47x10^{4}$	$1.64x10^{-4}$	-				
hMab5.5	VH2+VL1	$1.87x10^{-9}$	$9.05x10^{4}$	$1.69x10^{-4}$	++				
hMab5.6	VH2+VL2	$6.80x10^{-10}$	$9.88x10^{4}$	$6.72x10^{-5}$	+				
hMab5.7	VH2+VL3	$8.18x10^{-10}$	$8.13x10^{4}$	$6.65x10^{-5}$	+++				
**hMab5.8**	**VH2+VL4**	$\mathbf{7.13x10^{-10}}$	$\mathbf{1.05x10^{5}}$	$\mathbf{7.49x10^{-5}}$	**++++**	$\mathbf{3.8 \times 10^{-12}}$	$\mathbf{3.9 \times 10^{5}}$	$\mathbf{1.5 \times 10^{-6}}$	**59.7**
**hMab5.9**	**VH3+VL1**	$\mathbf{6.23x10^{-10}}$	$\mathbf{8.65x10^{4}}$	$\mathbf{5.39x10^{-5}}$	**++++**	$\mathbf{1.3 \times 10^{-10}}$	$\mathbf{4.6 \times 10^{5}}$	$\mathbf{5.9 \times 10^{-5}}$	**79.7**
**hMab5.10**	**VH3+VL2**	$\mathbf{4.06x10^{-10}}$	$\mathbf{9.12x10^{4}}$	$\mathbf{3.70x10^{-5}}$	**+++**	$\mathbf{9.5 \times 10^{-11}}$	$\mathbf{4.4 \times 10^{5}}$	$\mathbf{4.1 \times 10^{-5}}$	**72.9**
**hMab5.11**	**VH3+VL3**	$\mathbf{6.09x10^{-10}}$	$\mathbf{8.77x10^{4}}$	$\mathbf{5.34x10^{-5}}$	**++++**	$\mathbf{2.3 \times 10^{-11}}$	$\mathbf{4.5 \times 10^{5}}$	$\mathbf{1.0 \times 10^{-5}}$	**63.8**
hMab5.12	VH3+VL4	$5.74x10^{-10}$	$8.89x10^{4}$	$5.11x10^{-5}$	+++				
hMab5.13	VH4+VL1	$6.94x10^{-10}$	$8.34x10^{4}$	$5.79x10^{-5}$	+++				
hMab5.14	VH4+VL2	$7.69x10^{-10}$	$1.04x10^{5}$	$7.99x10^{-5}$	-				
**hMab5.15**	**VH4+VL3**	$\mathbf{6.81x10^{-10}}$	$\mathbf{9.14x10^{4}}$	$\mathbf{6.23x10^{-5}}$	**++++**	$\mathbf{8.4 \times 10^{-12}}$	$\mathbf{3.9 \times 10^{5}}$	$\mathbf{3. \times 10^{-6}}$	**47.3**
hMab5.16	VH4+VL4	$8.00x10^{-10}$	$1.08x10^{5}$	$8.66x10^{-5}$	++++				
**hMab5.17**	**VH5+VL1**	$\mathbf{3.68x10^{-10}}$	$\mathbf{1.03x10^{5}}$	$\mathbf{3.77x10^{-5}}$	**++++**	$\mathbf{1.3 \times 10^{-11}}$	$\mathbf{4.3 \times 10^{5}}$	$\mathbf{5.7 \times 10^{-6}}$	**12.2**

**hMab5.18**	**VH5+VL2**	**$4.26x10^{-10}$**	**$1.08x10^{5}$**	**$4.59x10^{-5}$**	**+++**	**2.2×10^{-11}**	**4.1×10^{5}**	**8.7×10^{-6}**	**34.1**
**hMab5.19**	**VH5+VL3**	**$6.02x10^{-10}$**	**$9.66x10^{4}$**	**$5.81x10^{-5}$**	**++++**	**2.8×10^{-11}**	**5.5×10^{5}**	**1.5×10^{-5}**	**22.2**
**hMab5.20**	**VH5+VL4**	**$4.98x10^{-10}$**	**$1.10x10^{5}$**	**$5.49x10^{-5}$**	**++++**	**2.3×10^{-10}**	**6.2×10^{5}**	**1.4×10^{-4}**	**44.1**

[A] The affinity of eukaryotic expression supernatant to rS21047-1210 was assessed using Biacore 8K, and the data were processed using Biacore 8K Evaluation software version 1.1. [B] The neutralization activity of mAbs in expression supernatant against authentic SARS-CoV-2 was analyzed by cytopathic effects (CPE) and summarized as "++++" (100%), "+++" (50-75%), "++" (25-50%), "+" (<25%), and "-" (no cytopathy). mAbs with good neutralizing capacity and binding affinity, which are shown in boldface type, were further expressed in CHO cells and purified for affinity and IC_{50} measurement. [C] Binding kinetics of purified mAb were determined by biolayer interferometry, and the source data are shown in Supplementary Figure S4.
